# Development of a novel liposomal nanodelivery system for bioluminescence imaging and targeted drug delivery in ErbB2-overexpressing metastatic ovarian carcinoma

**DOI:** 10.3892/ijmm.2014.1922

**Published:** 2014-09-04

**Authors:** XIAO-JIAN HAN, YONG-FANG WEI, YU-YING WAN, LI-PING JIANG, JIAN-FENG ZHANG, HONG-BO XIN

**Affiliations:** 1 Institute of Translational Medicine, Nanchang University, Nanchang, Jiangxi, P.R. China; 2 Department of Hospital Infection, The Second Affiliated Hospital of Nanchang University, Nanchang, Jiangxi, P.R. China; 3 Department of Pharmacology, Nanchang University School of Pharmaceutical Science, Nanchang, Jiangxi, P.R. China

**Keywords:** ErbB2, ovarian carcinoma, *Gaussia* luciferase, bioluminescence imaging, liposome, drug delivery system

## Abstract

Liposomes as targeted drug delivery systems are an emerging strategy in the treatment of cancer to selectively target tumors or genes. In this study, we generated the recombinant protein, EC1-GLuc, by fusing the EC1 peptide, an artificial ligand of ErbB2, with *Gaussia* luciferase (GLuc). The purified EC1-GLuc was conjugated with a nickel-chelating liposome to construct the EC1-GLuc-liposome. *In vitro* experiments revealed that the EC1-GLuc-liposome selectively targeted and internalized into ErbB2-overexpressing SKOv3 cells for bioluminescence imaging. A cell-impermeable fluorescence dye (HPTS) encapsulated in the EC-GLuc-liposome was efficiently delivered into the SKOv3 cells. In addition, the EC1-GLuc-liposome also targeted metastatic SKOv3 tumors for bioluminescence imaging and effectively delivered HPTS into metastatic tumors *in vivo*. Therefore, the present study demonstrates the novel EC1-GLuc-liposome to be an effective theranostic system for monitoring and treating ErbB2-overexpressing metastatic ovarian carcinoma through a combination of targeted molecular imaging and DDS.

## Introduction

ErbB2 is a member of the epidermal growth factor receptor (EGFR, also known as ErbB) family of receptor tyrosine kinases. There are 4 members in the ErbB family, including ErbB1-4. Endogenous ligands and the homo- or hetero-dimerization of ErbB receptors stimulate its tyrosine kinase activity, and regulate cellular proliferation and survival (1–3). Unlike other members of the ErbB family, ErbB2 is an orphan receptor without an endogenous ligand. However, the overexpression of ErbB2 is clinically associated with approximately 30% of ovarian cancers, breast cancers (4), and has been shown to correlate with the metastasis, therapeutic resistance and poor prognosis of cancer (5–7). In a phage display study, EC1, an artificial peptide, was found to bind the extracellular domain of ErbB2 in living cells and fresh-frozen human breast cancer specimens (8). Moreover, biotin-conjugated EC1 and the recombinant protein, EC1-eGFP, retained the affinity for ErbB2, and were selectively internalized into ErbB2-overexpressing cancer cells (8–10). Recently, divalent and multivalent forms of the EC1-Fc ligand in liposomes have been reported to improve the affinity for ErbB2 and enhance internalization (11). Thus, the EC1 peptide is a potential artificial ligand for targeting ErbB2.

On the other hand, bioluminescence has been extensively used as a relatively simple, cost-effective and extremely sensitive imaging system in intact cells and living animals (12). The most common luciferases for bioluminescence imaging include firefly luciferase (FLuc), *Renilla* luciferase (RLuc) and *Gaussia* luciferase (GLuc). Each luciferase has distinct properties in the application of bioluminescence imaging. FLuc (62 kDa) catalyzes the oxidation of luciferin to yield bioluminescence in the presence of O_2_, magnesium and adenosine triphosphate (ATP) (13). RLuc (36 kDa) and GLuc (19.9 kDa) catalyze the oxidative decarboxylation of coelenterazine (CTZ) to emit light independent of ATP. However, RLuc has a lower quantum yield than FLuc, and also less enzymatic efficiency (14,15). GLuc yields approximately 200- (*in vivo*) to 1,000-fold (*in vitro*) more bioluminescence in mammalian cells than FLuc and RLuc (16). The small molecular size (19.9 kDa), independence from ATP and strong emission render GLuc much more suitable for bioluminescence imaging (17,18).

Liposomes are mainly composed of phospholipids and cholesterols which are also the natural components in cells (19). The hydrophobic interactions lead to the self-assembly of lipid bilayers in water and form phospholipid vesicles (20). The concentric bilayers can be used to incorporate water-soluble materials, such as chemical compounds, proteins and siRNA in the aqueous core of the liposome (21–23). The addition of a conjugate of polyethylene glycol (PEG) linked to a lipid anchor to the liposomal formulation significantly prolongs the liposome circulation time, which greatly benefits the application of liposomes *in vivo* (24). Liposomes are nanoscale in size; thus, they are also considered as nanoscale drug delivery systems (DDS) or nanocapsules (25). In recent years, multifunctional liposomes have been developed to meet the requirements of different DDS by altering lipid composition, size and lipid surface modification (25,26). In our previous studies, the EGFR antibody-conjugated liposomes were constructed for the selective delivery of sodium borocaptate and bioluminescence imaging in EGFR-overexpressing glioma (18,22). In the present study, the EC1-GLuc-liposome was constructed to combine bioluminescence imaging and targeted DDS for ErbB2-overexpressing metastatic ovarian carcinoma.

Ovarian carcinoma is one of the most common causes of mortality from gynaecological malignancies. Ovarian carcinoma is highly metastatic, and patients mainly succumb to the disease due to metastasis (27). Unlike other solid tumors, the metastasis of ovarian carcinoma is mostly induced by direct dissemination into the peritoneal cavity, rather than circulatory or lymphatic metastasis (28). Due to the complexity of the peritoneal cavity, it is difficult to clean-up the metastatic foci using currently available therapeutic strategies, such as surgery, radiotherapy and chemotherapy. Therefore, novel therapeutic strategies involving targeted molecular imaging and DDS are urgently required in order to improve the therapeutic efficacy in metastatic ovarian carcinoma. In this study, a novel ErbB2-targeting bioluminescence protein was generated by fusing EC1 with GLuc, and was conjugated to a liposome to construct the EC1-GLuc-liposome. MCF7 (ErbB2-negative) and SKOv3 (ErbB2-overexpressing) cells were employed to evaluate the efficacy of the EC1-GLuc-liposome in ErbB2-targeted imaging and drug delivery. It was found that the ErbB2-targeted bioluminescence imaging and selective delivery of HPTS by the EC1-GLuc-liposome was effective not only in the SKOv3 cells *in vitro*, but also in metastatic ovarian tumors *in vivo*. Thus, the present study suggests the EC1-GLuc-liposome to be a novel strategy for combining bioluminescence imaging and drug delivery for monitoring and treating ErbB2-overexpressing metastatic ovarian carcinoma.

## Materials and methods

### Cell culture and detection of ErbB2 expression

The MCF7 and SKOv3 cell lines were obtained from the American Type Culture Collection (ATCC, Manassas, VA, USA). The 2 cell lines were cultured in Dulbecco’s modified Eagle’s medium (DMEM) medium supplemented with 10% fetal bovine serum (FBS) and 100 μg/ml penicillin-streptomycin (P/S). The medium, FBS and P/S were from Invitrogen (Carlsbad, CA, USA). All cultures were maintained in a humidified incubator at 37°C with an atmosphere containing 5% CO_2_.

The expression of ErbB2 in the MCF7 and SKOv3 cells was examined by immunoblot analsyis. Briefly, cells on 35 mm dishes were washed once with phosphate-buffered saline (PBS), and scraped in 1X SDS sample buffer. Cell lysates were subjected to 6% SDS-PAGE, and immunoblotted with rabbit anti-ErbB2 antibody (1:1,000; Cell Signaling Technology, Danvers, MA, USA) overnight at 4°C. HRP-conjugated secondary antibody (Sigma-Aldrich, St. Louis, MO, USA) was used at 1:2,000. Immunoblotting signals were detected using the VersaDoc 5000 Imaging system (Bio-Rad, Hercules, CA, USA) with an enhanced chemiluminescence detection kit (Amersham Biosciences, Pittsburgh, PA, USA).

### Plasmid construction

The pGLuc plasmid was purchased from LUX Biotechnology Ltd. (Scotland, UK). The GLuc gene was amplified from the plasmid using a sense primer (5′-GGATCCG AAACCAACTGAAAACAATGAAG-3′) and an antisense primer (5′-GTCGACATCACCACCGGCACCCTTTAT-3′). The PCR product was ligated into the pCR2.1 TOPO vector (Invitrogen) for amplification, and recloned in the pET52b(+) vector (Invitrogen) to construct GLuc-pET52b(+). The following sense and antisense oligonucleotides with appropriate resistance enzyme sites for EC1 peptide were prepared: 5′-GGGTGGAC TGGCTGGTGCCTGAATCCAGAAGAATCTACTTGGGG ATTCTGTACTGGATCTTTCGGTGGAGGTAGTTCAG-3′ (sense, underline indicates *Sam*I and *Bam*HI sites), 5′-GATCCT GAACTACCTCCACCGAAAGATCCAGTACAGAATCCCC AAGTAGATTCTTCTGGATTCAGGCACCAGCCAGTCCA CCC-3′ (antisense, underline indicates *Bam*HI and *Sam*I sites). The oligonucleotides for EC1 were modified by phosphorylation at 5′. The sense and antisense oligonucleotides (10 pmol each) were co-incubated at 95°C for 10 min, and annealed at room temperature to form double-stranded DNA. The double-stranded DNA for EC1 was ligated to GLuc-pET52b treated with the appropriate resistance enzymes to construct EC1-GLuc-pET52b. The sequences of the constructed plasmids were confirmed using an ABI 3100 sequencer (Applied Biosystems, Bedford. MA, USA).

### Expression and purification of recombinant proteins

Recombinant protein expression and purification was carried out as previously described (10). Briefly, *E. coli* BL21 (DE3) transformed with the plasmid for GLuc or EC1-GLuc was grown in a LB medium containing 100 μg/ml ampicillin at 37°C. When the OD_600_ reached 0.6, the expression of the recombinant proteins was induced by the addition of 0.2 mM isopropyl 1-thio-*β*-D-galactopyranoside at 25°C overnight. The GLuc-6His and EC1-GLuc-6His proteins were purified from the supernatant using ProBond Nickel-chelating resin (Invitrogen). The recombinant proteins were subjected to 15% SDS-PAGE, and the expression was confirmed by Coomassie brilliant blue staining and western blot analysis with rabbit anti-GLuc serum (1:1,000; Nanolight Technology, Pinetop, AZ, USA). The proteins were finally dialyzed against PBS (pH 7.4) at 4°C for 24 h, and the concentrations were determined using a Biadford protein assay kit (Bio-Rad). Aliquots of protein were stored at −80°C prior to use.

### Construction of GLuc-liposome and EC1-GLuc-liposome

Liposomes composed of DOPC:DOPG:DOGS-NTA-Ni:CH:DSPE-PEG_2000_ (molar ratio, 3:3:1:4:0.1) were prepared as previously described with a slight modification (18,22). Briefly, 100 μM of lipid were dissolved in 2 ml of a chloroform/diethyl ether mixture (1:1 v/v). The ratio of organic to aqueous phase was 2:1. The mixture was added to a rotary evaporator to form a lipid gel at 50°C under reduced pressure. Subsequently, 2 ml of 35 mM HPTS in PBS was mixed with the lipid gel. The mixture was vortexed and sonicated. To obtain liposomes with homogeneous size, the liposome emulsion was extruded through a polycarbonate membrane 100 nm in pore size using an extruder device at 60°C. The mean diameter and zeta potential of the prepared liposomes were determined using an electrophoretic light scattering spectrophotometer (ELS-8000; Photal, Osaka, Japan). The unencapsulated free HPTS was removed using a PD-10 desalting column (Amersham Biosciences). To construct luciferase-conjugated liposomes, the nickel-chelated liposome was incubated with GLuc-His or EC1-GLuc-His at a molar ratio of 20:1 overnight at room temperature at a low rotating speed. Free GLuc-His or EC1-GLuc-His was removed with a sepharose CL-4B column and the eluted GLuc or EC1-GLuc conjugated liposomes were concentrated. The lipid in each fraction was analyzed by the DAOS method using a Phospholipid C reagent kit (Wako Pure Chemical Industiries, Ltd., Osaka, Japan). The recombinant protein conjugated on the liposome in each fraction was detected by western blot analayis with rabbit anti-GLuc serum or a Biadford protein assay kit.

### Preparation of CTZ

The substrate for GLuc was prepared as previously described (10,12). Briefly, CTZ (Nanolight Technology) was dissolved in acidified methanol [1 drop of concentrated HCl (12.4 N) in 10 ml of methanol] to a concentration of 5 mg/ml. Aliquots of 100 μl were stored at −80°C. For luminescence imaging, the aliquots of CTZ (5 mg/ml) were diluted with PBS (containing 5 mM NaCl, pH 7.2) for greater light output and more stability.

### Bioluminescence imaging and selective delivery of HPTS into ErbB2-overexpressing cells in vitro by the EC1-GLuc-liposome

For bioluminescence imaging *in vitro*, the MCF7 and SKOv3 cells were cultured on 35-mm glass-bottomed dishes for 24–48 h. To improve the adherence of MCF7 and SKOv3 cells, all glass-bottomed dishes were pre-coated with laminin (Roche Diagnostics, Indianapolis, IN, USA). The MCF7 and SKOv3 cells were incubated with 1 mM GLuc-liposome or EC1-GLuc-liposome. After 2 h of incubation, the cells were washed 3 times with DMEM. Bioluminescence was detected with an Olympus Luminoview LV 200 bioluminescence microscope (Olympus, Tokyo, Japan) immediately after the addition of 1 μg/ml CTZ to the serum-free DMEM. Images were acquired and analyzed using Metamorph software (Molecular Devices, Sunnyvale, CA, USA).

To examine the internalization of the luciferase-conjugated liposomes in cells, the MCF7 and SKOv3 cells were incubated with 1 mM of the liposomes. In a blocking experiment, the SKOv3 cells were treated with anti-ErbB2 antibody against the extracellular domain of ErbB2 (chicken, 1.0 μg/ml; Abcam, Cambridge, MA, USA) 1 h prior to incubation with EC1-GLuc-liposome. After 2 h of incubation, the cells were washed with PBS 3 times and trypsinized. For western blot analysis, cell lysate was prepared and subjected to 15% SDS-PAGE. The internalization of the luciferase-conjugated liposomes was analyzed by immunoblotting with rabbit anti-GLuc serum. β-actin was used as an endogenous control. To detect the delivery of HPTS, the cells were fixed with 4% paraformaldehyde (PFA) for 10 min, and washed with PBS twice. The fluorescence signal of HPTS in the cells was observed using a confocal laser microscope (Olympus).

### Preparation of tumor-bearing mice

All animal handling was performed in accordance with the Animal Research Committee guidelines of Nanchang University, Nanchang, China. The cultured SKOv3 cells were washed twice with PBS, typsinized and harvested by centrifugation. The SKOv3 cells (5.0×10^6^) suspended in DMEM were intraperitoneally implanted in anesthetized nude mice (Balb/c Slc-nu/nu, female, 6–8 weeks old) under aseptic conditions. After cell implantation, the mice with tumors which had developed for 3 weeks were used for *in vivo* experiments. During tumor development, breath and behavioral activity were monitored twice each day to evaluate pain in the mice. The experiments were immediately terminated if the mice presented with significant pain symptoms. All mice were euthanized by an intraperitoneal injection of 15 mg/100 g pentobarbital solution after the experiments.

The expression of ErbB2 in the SKOv3 tumor xenografts was confirmed by immunofluorescence (IF) staining. Paraffin-embedded slices of tumors were prepared at a thickness of 5 μm. First, histological observations of the xenografted tumors were made using hematoxylin and eosin (H&E) staining. IF staining was performed with anti-ErbB2 antibody (1:50; Cell Signaling Technology). The secondary antibody was Cy3-conjugated anti-rabbit IgG (1:100; Invitrogen, Molecular Probes). The nucleus was counterstained with 0.1 μg/ml of Hoechst 33248 (Sigma-Aldrich) for 5 min. Fluorescence signals were observed using a confocal laser microscope (FV500; Olympus).

### Bioluminescence imaging and delivery of HPTS into metastatic SKOv3 tumors in vivo by the EC1-GLuc-liposome

The mice bearing metastatic SKOv3 tumors were administrated intravenously 300 μl of luciferase-conjugated liposomes (40 mM) via the tail vein. After 8 h, the mice were imaged under anesthesia by intraperitoneally injecting 200 μl of CTZ solution (5 mg/kg) using a cooled CCD camera (IVIS; Xenogen, Alameda, CA, USA) as previously described (18). In addition, a laparotomy was performed to directly image the SKOv3 tumors using the cooled CCD camera. The bioluminescence intensity of the selected region over the tumor was recorded as maximum photons sec/cm^2^/steradian. To detect the delivery of HPTS *in vivo* by the EC1-GLuc-liposome, the mice were euthanized by an intraperitoneal injection of 15 mg/100 g pentobarbital solution soon after bioluminescence imaging. The metastatic SKOv3 tumors were removed for preparation of 10-μm tissue section. The fluorescence signal of HPTS was observed using a confocal laser microscope (Olympus).

## Results

### Construction and characterization of the EC1-GLuc-liposome

**Figure 1:**
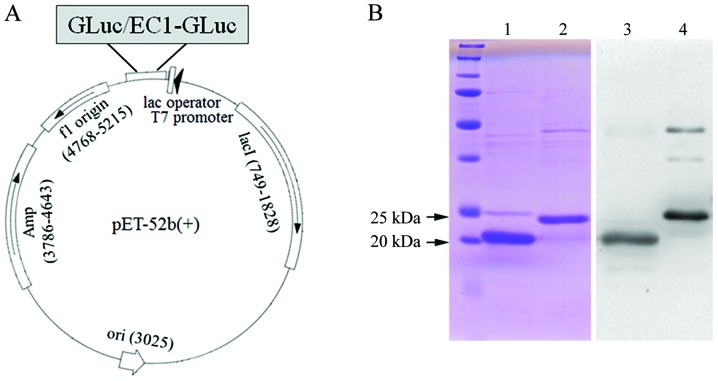
Purification of the recombinant proteins. (A) Diagram of the subclone of *Gaussia* luciferase (GLuc) and EC1-GLuc into the pET-52b(+) vector. (B) Coomassie brilliant blue staining and western blot analysis of purified proteins. Lanes 1 and 2, Coomassie brilliant blue staining of GLuc and EC1-GLuc, respectively. Lanes 3 and 4, western blot analysis of GLuc and EC1-GLuc, respectively.

**Figure 2:**
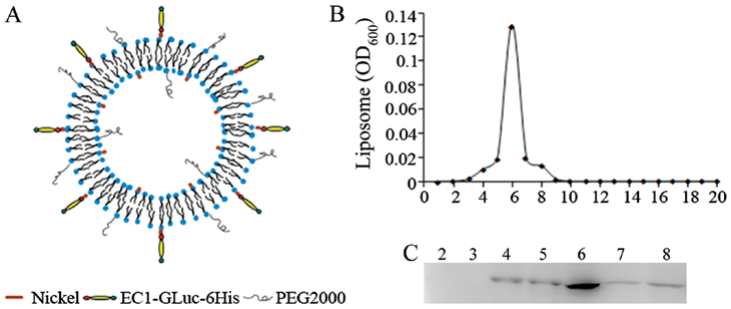
Characterization of EC1-*Gaussia* luciferase (GLuc)-conjugated liposomes. (A) Schematic diagram of components of the liposome. (B) Preparation of the EC1-GLuc-liposome. The liposome was eluted through a sepharose column, and phospholipids in different fractions were measured by the DAOS method. The absorbance at OD_600_ was used to indicate the position of the liposome. (C) Identification of the eluted EC1-GLuc-liposome. EC1-GLuc conjugated to the liposome in different fractions was detected by western blot analysis with anti-GLuc serum.

Firstly, GLuc and EC1-GLuc were subcloned into the pET-52b(+) vector for the expression of recombinant proteins (Fig. 1A). The recombinant GLuc-His and EC1-GLuc-His were expressed in *E. coli* BL21 and purified using a column of ProBond Nickel-chelating resin. Following purification, the recombinant proteins were subjected to 15% SDS-PAGE and confirmed with Coomassie brilliant blue staining and western blot analysis (Fig. 1B). The results reveaked that GLuc-His and EC1-GLuc-His were highly purified. The molecular sizes of GLuc-His and EC1-GLuc-His were approximately 20 and 23 kDa, respectively. On the other hand, the molar ratio of DOPC:DOPG:DOGS-NTA-Ni:CH:DSPE-PEG_2000_ in 3:3:1:4:0.1 was used as the nickel-liposome formulation (Table I). A cell-impermeable fluorescence dye (HPTS) was encapsulated in the liposomes as previously described (18). The recombinant proteins were conjugated to the liposomes through their affinity with nickel to construct the GLuc-liposome and EC1-GLuc-liposome (Fig. 2A). The GLuc-liposome was used as the control in the experiments. After construction of the luciferase-conjugated liposomes, the diameter and zeta potential of the liposomes were measured (Table I). The diameter of the liposomes was 106.37 to 117.58 nm. The zeta potential was −30.46 to −27.52 mV. To examine the conjugation of EC1-GLuc-His to the nickel-liposome, the positions of the recombinant protein and liposome were compared after their separation with Sepharose CL-4B. The liposome mostly appeared in fraction 6 (Fig. 2B). Western blot analysis indicated that EC1-GLuc-His was also most abundant in fraction 6 (Fig. 2C). These results suggest that EC1-GLuc-His was effectively conjugated to the surface of the liposome through its affinity with nickel.

### ErbB2-targeting and delivery of HPTS by the EC1-GLuc-liposome in vitro

**Figure 3:**
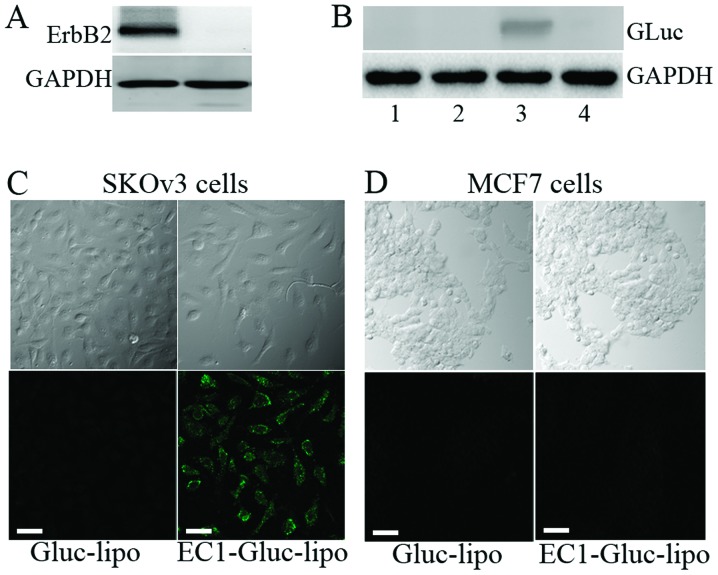
ErbB2-targeted internalization and delivery of HPTS into cells by the EC1-*Gaussia* luciferase (GLuc)-liposome. (A) Expression of ErbB2 in MCF7 and SKOv3 cells. (B) The selective internalization of EC-GLuc-liposome into SKOv3 cells. Cells were incubated with GLuc- or EC1-GLuc-conjugated liposomes for 2 h. Lane 1, EC1-GLuc-liposome (MCF7); lane 2, GLuc-liposome (SKOv3); lane 3, EC1-GLuc-liposome (SKOv3); lane 4, anti-ErbB2 + EC1-GLuc-liposome (SKOv3). After washing and trypsinization, the internalization of the liposome was analyzed by immunoblotting with rabbit anti-GLuc serum. Glyceraldehyde 3-phosphate dehydrogenase (GAPDH) was used as an endogenous control. (C and D) Delivery of the green fluorescence dye, HPTS, into SKOv3 and MCF7 cells by GLuc-liposome or EC1-GLuc-liposome. The upper panels are phase-contrast images, the lower panels are fluorescence images. (C) SKOv3 and (D) MCF7 cells were treated with the GLuc-liposome or EC1-GLuc-liposome respectively. Green fluorescence of HPTS was only detected in SKOv3 cells treated with the EC1-GLuc-liposome. Scale bar, 50 μm.

Two human carcinoma cell lines, MCF7 and SKOv3, were used for the *in vitro* experiments. Firstly, the expression level of ErbB2 in the 2 cell lines was examined by western blot analysis. The overexpression of ErbB2 was observed in the SKOv3 cells, while the expression of ErbB2 was undetectable in the MCF7 cells (Fig. 3A). Moreover, western blot analysis revealed that the internalization of the recombinant protein was only observed in the EC1-GLuc-liposome treated SKOv3 cells. The internalization was mostly undetectable when the extracellular domain of ErbB2 was blocked by anti-ErbB2 antibody (Fig. 3B). The selective delivery of HPTS was also observed in the SKOv3 cells treated with the EC1-GLuc-liposome, but not in the MCF7 cells (Fig. 3C). These results suggest that the EC1-GLuc-liposome retains its ErbB2-targeting and is effective for DDS *in vitro*.

### The EC1-GLuc-liposome targets ErbB2 for bioluminescence imaging in vitro

**Figure 4:**
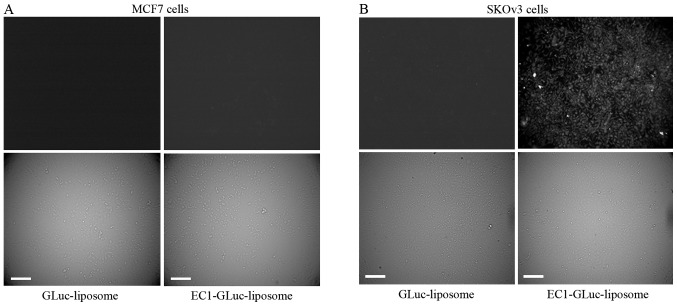
ErbB2-targeted bioluminescence imaging of EC1-*Gaussia* luciferase (GLuc)-liposome *in vitro*. (A) MCF7 and (B) SKOv3 cells were incubated with the GLuc-liposome or EC1-GLuc-liposome for 2 h. The upper panels are bioluminescence images, and the bottom panels are phase-contrast images. The bioluminescence signal was observed in the SKOv3 cells incubated with the EC1-GLuc-liposome, but not in the other experimental groups. Scale bar, 100 μm.

To detect the ErbB2-targeted bioluminescence, the MCF7 and SKOv3 cells were incubated with 1 mM GLuc-liposome or EC1-GLuc-liposome. After 2 h of incubation, the cells were washed 3 times with DMEM. The bioluminescent signal was detected using an Olympus Luminoview LV 200 bioluminescence microscope immediately after the addition of 1 μg/ml CTZ in serum-free DMEM. A strong signal was detected in ErbB2-overexpressing SKOv3 cells incubated with the EC1-GLuc-liposome (Fig. 4). By contrast, no significant bioluminescence was observed in the SKOv3 cells incubated with the GLuc-liposome and the MCF7 cells incubated with both liposomes. This indicates that the EC1-GLuc-liposome effectively targets ErbB2-overexpressing cells for bioluminescence imaging *in vitro*.

### Bioluminescence imaging and delivery of HPTS into metastatic ovarian carcinoma tumors by the EC1-GLuc-liposome in vivo

**Figure 5:**
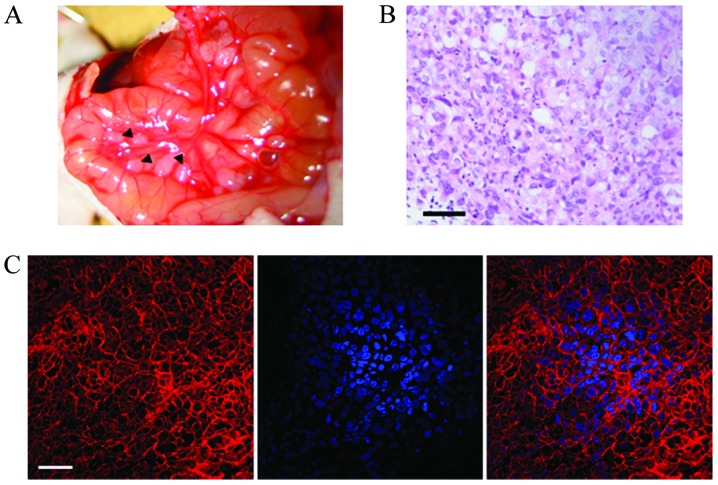
Preparation of metastatic SKOv3 tumor-bearing mice. (A) Observation of metastatic SKOv3 tumors in mice. Three weeks after the intraperitoneal implantation of 5×10^6^ SKOv3 cells, a laparotomy was performed to observe metastatic SKOv3 tumors in mice. The black triangular arrows indicate the metastatic SKOv3 tumors. (B) Histological observation of metastatic SKOv3 tumors using hematoxylin and eosin (H&E) staining. Scale bar, 100 μm. (C) Expression of ErbB2 in metastatic ovarian tumors derived from SKOv3 cells. Paraffin-embedded slices of tumors were subjected to immunofluorescence (IF) staining with rabbit anti-ErbB2 antibody and Hoechst 33248 for nuclear staining. Scale bars, 50 μm.

**Figure 6:**
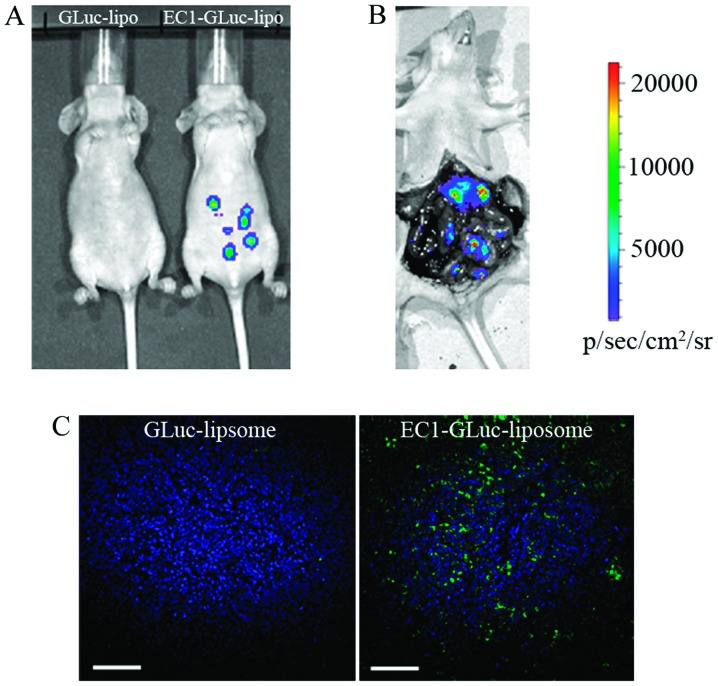
Bioluminescence imaging and delivery of HPTS into metastatic SKOv3 tumors by EC1-*Gaussia* luciferase (GLuc)-liposome. (A) Bioluminescence imaging of metastatic SKOv3 tumor-bearing mice. A GLuc-liposome-treated mouse is shown on the left. AEC1-GLuc-liposome-treated mouse is shown on the right. (B) Bioluminescence imaging of metastatic SKOv3 tumor-bearing mice after laparotomy. The color scale represents p/sec/cm^2^/steradian. (C) Delivery of HPTS into metastatic ovarian tumors by the EC1-GLuc-liposome. Green represents the fluorescence of HPTS delivered into the tumor, and blue represents the nuclear staining with Hoechst 33248. The image on the left is from a tumor section treated with the GLuc-liposome, and the one on the right is from a tumor section treated with the EC1-GLuc-liposome. Scale bars, 100 μm.

For *in vivo* experiments, we established metastatic ovarian carcinoma tumor-bearing mice by the intraperitoneal implantation of 5×10^6^ SKOv3 cells. Following tumor development for 3 weeks, the metastatic ovarian tumors were clearly visible by laparotomy (Fig. 5A). Histological observations of the tumors were carried out using H&E staining (Fig. 5B). In addition, the expression of ErbB2 in the metastatic SKOv3 tumors was also confirmed by IF staining (Fig. 5C). To evaluate the ErbB2-targeted imaging of the EC1-GLuc-liposome *in vivo*, metastatic SKOv3 tumor-bearing mice were injected with the GLuc-liposome or EC1-GLuc-liposome via the tail vein. Eight hours later, bioluminescence images were acquired after the administration of CTZ. A strong bioluminescent signal was detected in the mice injected with the EC1-GLuc-liposome, while it was undetectable in the mice injected with the GLuc-liposome (Fig. 6A). Moreover, direct bioluminescence imaging was achieved after laparotomy in the metastatic SKOv3 tumor-bearing mice injected with the EC1-GLuc-liposome (Fig. 6B). To investigate the selective delivery of HPTS, the metastatic SKOv3 tumors were removed for section soon after bioluminescence imaging. A strong fluorescence signal of HPTS was observed in the EC1-GLuc-liposome-treated mice (Fig. 6C). However, in the GLuc-liposome-treated mice, no significant fluorescence was detected. These results suggest that the EC1-GLuc-liposome is effective for ErbB2-targeted bioluminescence imaging and targeted DDS for metastatic ovarian carcinoma *in vivo*.

## Discussion

The overexpression of certain cancer-specific surface molecules and gene mutations has been identified in cancer cells. Among these, the ErbB/EGFR family is one of the best known. On the other hand, some cancer-specific surface molecules have been successfully applied in targeted cancer therapy and imaging (30,31). In our previous study, we constructed a ErbB2-targeting protein by fusing the EC1 peptide with GLuc and the p53C peptide. The EC1-GLuc-p53C exerts its function for ErbB2-targeted bioluminescence and cancer therapy *in vitro*. However, the function of EC1-GLuc-p53C *in vivo* was very poor due to the very low accumulation in tumors when intravenously injected (10). In the present study, EC1-GLuc-liposome was constructed by conjugation EC1-GLuc-His to a nickel-chelating liposome. *In vitro* experiments indicated that the EC1-GLuc-liposome was selectively internalized and delivered HPTS into ErbB2-overexpressing SKOv3 cells (Fig. 3B and C). ErbB2-targeted bioluminescence imaging was carried out successfully in SKOv3 cells (Fig. 4). In addition, the bioluminescence imaging and selective delivery of HPTS into metastatic SKOv3 tumors *in vivo* were also achieved using the EC1-GLuc-liposome (Fig. 6). Thus, the *in vitro* and *in vivo* experiments in the present study demonstrated that the novel target liposome was effective for ErbB2-targeted bioluminescence imaging and drug delivery.

In our previous studies, the immunoliposomes were constructed by conjugating anti-EGFR antibody to liposomes using the antibody affinity motif of protein A (ZZ) as an adaptor for targeted DDS and the imaging of glioblastoma (18,22). These immunoliposomes effectively targeted EGFR-overexpressing glioblastoma cells for the delivery of borocaptate and bioluminescence imaging *in vitro* and *in vivo*. However, the immunoliposome contains 3 main components: the liposome, the ZZ protein and EGFR antibody. In the present study, the target liposome only contained only 2 components: the liposome and a small protein, EC1-GLuc. The decrease in components may facilitate the construction of the target liposome and may also decrease the size of the liposome. The diameter of the previous immunoliposome was approximately 130 nm (22), but the diameter of the EC1-GLuc-liposome was decreased to 117 nm (Table I). The smaller size may improve the drug delivery efficience of liposomes into targeted cells (32). In addition, it has been reported that divalent or multivalent forms of the EC1 peptide fused-Fc-liposome selectively target ErbB2-overexpressing breast cancer cells and are efficiently internalized into cells (11). Consistent with the previous studies, thye EC1-GLuc-liposome retains its affinity with ErbB2, and is effectively internalized into ErbB2-overexpressing SKOv3 cells for bioluminescence imaging and drug delivery.

Ovarian carcinoma is one of the common gynaecological tumors with a high metastatic rate (27,29). The metastatic foci of ovarian carcinoma in the peritoneal cavity are very difficult to be clean-up with conventional surgery, radiotherapy and chemotherapy (33). As a result, patients with ovarian carcinoma mainly succumb to the disease due to metastasis. Therefore, a reliable approach for visualizing metastatic ovarian tumors and a targeted DDS are urgently required in order improve the therapeutic effects. In the present study, the EC1-GLuc-liposome selectively targeted ErbB2 for bioluminescence imaging and drug delivery for metastatic ovarian carcinoma *in vitro* and *in vivo*. Therefore, the EC1-GLuc-liposome may not only service as a surgical navigation to clean-up metastatic ovarian carcinoma by bioluminescence imaging, but may also provide a molecular targeting DDS to improve the chemotherapy and radiotherapy in the future.

The combination of a diagnostic test and a therapeutic entity is termed theranostics (34,35). In recent years, theranostics has developed rapidly along with selective targeting strategies. The targeting moieties include proteins (mainly antibodies and their fragments), peptides and some small molecules (folate and flavin mononucleotide) (35). Peptides are attractive targeting molecules due to their small size, low immunogenicity and ease of manufacture at low costs. In the present study, we used the EC1 peptide as a targeting moiety. The EC1-GLuc-liposome effectivly targeted ErbB2-overexpressing ovarian cancer cells for bioluminescence imaging *in vitro* and *in vivo*, and also proved to be effective in targeted DDS. Thus, our multifunctional EC1-GLuc-liposome may prove to be a promising theranostic reagent for ErbB2-overexpressing metastatic ovarian carcinoma in the future, although the development of this technology is still at the early stage and requires some optimization.

## Figures and Tables

**Table I:** Composition and properties of liposomes.

Lipid composition (mmol)	DOPC	30
	DOPG	30
	Chol	40
	DOGS-NTA-Ni	10
	DSPE-PEG_2000_	1
	Total (mmol)	111
Lipid properties	zeta potential (mV)	−30.46 to 27.52
	Size (nm)	106.37 to 117.58

DOPC, 1,2-dioleoyl-sn-glycero-3-phosphocholine; DOPG, 1,2-dioleoyl- sn-glycero-3-phosphoglycerol; Chol, cholesterol; DOGS-NTA-Ni, 1,2-di-(9Z-octadecenoyl)-sn-glycero-3-[(N-(5-amino-1-carboxypentyl)iminodiacetic acid)succinyl] (nickel salt); DSPE-PEG_2000_, 1,2-distearoyl-sn-glycero-3-phosphoethanolamine-N-[methoxy(polyethylene glycol)-2000].
